# Supplementary material for: Identification of photoperiod-induced specific miRNAs in the adrenal glands of Sunite sheep (Ovis aries)
Source: Front Vet Sci. 2022 Jul 22;9:888207. doi: 10.3389/fvets.2022.888207 (PMC9354845; doi:10.3389/fvets.2022.888207)
Supplement: Supplementary file 1 [file Data_Sheet_1.ZIP › Supplementary file/miRDeep2 file.zip!/miRDeep2 file/Novel_Structure/SPLP42A3/Novel_150.pdf]

Provisional ID	:	NC_019484.2_39251
Score total	:	2.7
Score for star read(s)	:	-1.3
Score for read counts	:	0
Score for mfe	:	2.4
Score for randfold	:	1.6
Score for cons. seed	:	
Total read count	:	441
Mature read count	:	437
Loop read count	:	0
Star read count	:	4

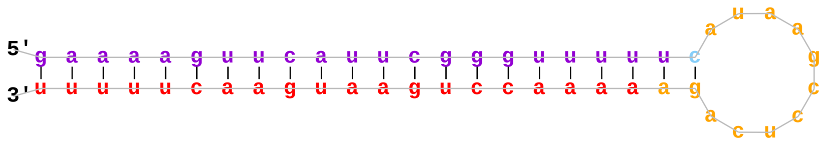

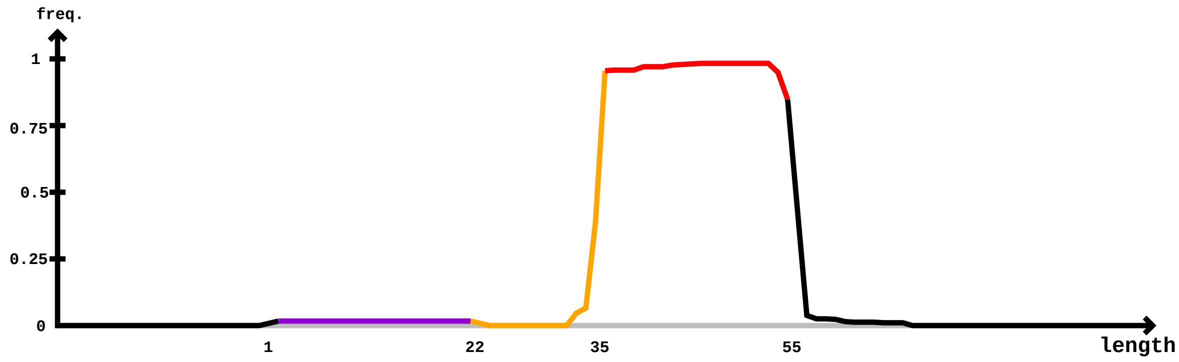

Star Mature

```
5'- cuaucauauauuggguuggcugaaaaguucauucggguuuuucauaagccucagaaaaaccugaaugaacuuuuuggccaacccaauauuaucuaacuaucccaguggaguc  -3'  obs
    cuaucauauauuggguuggcugaaaaguucauucggguuuuucauaagccucagaaaaaccugaaugaacuuuuuggccaacccaauauuaucuaacuaucccaguggaguc        exp
    .......((((((((((((((((((((((((((((((((((((..........))))))))))))))))))))))))))))))))))))..........(((....)))...   reads  mm  sample
    ....................ugaaaaguucauucggguuuuu......................................................................   4      0   seq
    .....................gaaaaguucauucggguuuuu......................................................................   4      0   seq
    ....................................................agaaaaaUcugaaugaacuuu.......................................   1      1   seq
    ....................................................agaaaaaUcugaaugaacuuuu......................................   3      1   seq
    ....................................................agaaaaaccugaaugaacuuuu......................................   10     0   seq
    ....................................................agaaaaaUcugaaugaacuuuuu.....................................   8      1   seq
    .....................................................gaaaaaccCgaaugaacuuu.......................................   1      1   seq
    .....................................................gaaaaaccAgaaugaacuuuu......................................   1      1   seq
    .....................................................gaaaaaccugaaugaacuuuu......................................   4      0   seq
    .....................................................gaaaaaccCgaaugaacuuuu......................................   1      1   seq
    .....................................................gGaaaaccugaaugaacuuuuu.....................................   1      1   seq
    .....................................................gaaaaaccugaaCgaacuuuuu.....................................   1      1   seq
    ......................................................aaaaaUcugaaugaacuuu.......................................   12     1   seq
    ......................................................aaaaaccCgaaugaacuuu.......................................   1      1   seq
    ......................................................aaaaaccugaaugaacCuu.......................................   1      1   seq
    ......................................................aaaaaUcugaaugaacuuuu......................................   26     1   seq
    ......................................................aaaaaccugaaGgaacuuuu......................................   1      1   seq
    ......................................................aaaaaccugaaugaacCuuuu.....................................   1      1   seq
    ......................................................Gaaaaccugaaugaacuuuuu.....................................   1      1   seq
    ......................................................aaaaaUcugaaugaacuuuuu.....................................   78     1   seq
    ......................................................aaaaaUcugaaugaacuuuuug....................................   24     1   seq
    ......................................................aaaaaccugaaCgaacuuuuug....................................   3      1   seq
    ......................................................aaaaaccCgaaugaacuuuuug....................................   2      1   seq
    ......................................................aaaaaccugaaugaacCuuuug....................................   1      1   seq
    ......................................................aaaaaccugaaugaacuuuuugg...................................   2      0   seq
    .......................................................aaaaccugaaugGacuuuu......................................   1      1   seq
    .......................................................aaaaccAgaaugaacuuuu......................................   1      1   seq
    .......................................................aaaaccAgaaugaacuuuuu.....................................   2      1   seq
    .......................................................aaaaUcugaaugaacuuuuu.....................................   247    1   seq
    .......................................................aaaaccugaaugaacuuuuu.....................................   8      0   seq
    .......................................................aaaCccugaaugaacuuuuu.....................................   1      1   seq
    .......................................................aaaaccugaaugaacuuuuuU....................................   2      1   seq
    .......................................................Gaaaccugaaugaacuuuuug....................................   1      1   seq
```

Star **Mature**

```
cuaucauauauuggguuggcugaaaaguucauucggguuuuucauaagccucagaaaaaccugaaugaacuuuuuggccaacccaauauuaucuaacuaucccaguggaguc
.......................................................aaaCccugaaugaacuuuuug....................................   2   1   seq
.......................................................aaaaccugGaugaacuuuuugg...................................   1   1   seq
.......................................................Gaaaccugaaugaacuuuuugg...................................   2   1   seq
.......................................................aaaaccGgaaugaacuuuuugg...................................   1   1   seq
........................................................aaaccAgaaugaacuuuuug....................................   1   1   seq
...........................................................ccugaaugaacuuuuuggcc.................................   1   0   seq
...........................................................ccugaaugaacuuuuuggcca................................   4   0   seq
...........................................................ccugaaugaacuuuuuggccaa...............................   1   0   seq
..............................................................gaaugaacuuuuuggccaacccaUu.........................   3   1   seq
...............................................................aaugaacuuuuuggccaaccc............................   1   0   seq
................................................................augaacuuuuuggccaacccaaA.........................   1   1   seq
.................................................................ugaacuuuuuggccaacccaau.........................   1   0   seq
```
